# Analysis of left ventricular function, left ventricular outflow tract and aortic valve area using computed tomography: Influence of reconstruction parameters on measurement accuracy

**DOI:** 10.1259/bjr.20201306

**Published:** 2021-07-08

**Authors:** Michaela M Hell, Bettine Steinmann, Tassilo Scherkamp, Martin B Arnold, Stephan Achenbach, Mohamed Marwan

**Affiliations:** 1 Department of Cardiology, Friedrich-Alexander-University of Erlangen-Nürnberg, Erlangen, Germany; 2 Department of Cardiology, University Medical Center Mainz, Johannes Gutenberg University, Mainz, Germany

## Abstract

**Objectives:**

Computed tomography (CT) allows reproducible assessment of left ventricular (LV) function, left ventricular outflow tract area (LVOT_area_) and aortic valve area (AVA). We evaluated the influence of image reconstruction parameters on these measurements.

**Methods:**

We analyzed 45 contrast-enhanced, retrospectively ECG-gated CT datasets acquired on a third-generation dual source system. A standard filtered-back-projection data set (20 cardiac phases (5% steps, 0–95%), 0.6-mm-slice thickness, 512 × 512 matrix) and eight reconstructions with modified slice thickness (1–8 mm), number of cardiac phases (5, 10), matrix size (256×256) and an iterative reconstruction (IR) algorithm were obtained. LV parameters (ejection fraction (EF), stroke volume (SV), end-diastolic (EDV), end-systolic volumes (ESV)), LVOT_area_ and AVA were assessed.

**Results:**

Differences in LV parameters, LVOT_area_ and AVA, were only minimal between standard reconstructions and those with modified matrix size, IR algorithm and ≤2 mm slice thickness, while reconstructions with 8-mm slice thickness significantly overestimated SV (*p* < 0.001) and EDV (*p* = 0.016). AVA planimetry in reconstructions with ≥5 mm slice thickness was not feasible in 56% of patients. A decrease in the number of reconstructed phases (10 or 5) underestimated EF, SV, EDV, LVOT_area_ and AVA and overestimated ESV.

**Conclusions:**

Modifications of reconstruction parameters (except a slice thickness ≤2 mm) have only a marginal effect on LV, LVOT_area_ and AVA assessment. However, a reduced number of reconstructions per cardiac cycle may significantly influence measurements.

**Advances in knowledge:**

Substantial modifications in number of reconstructions per cardiac cycle significantly affect the assessment of LV function, LVOT_area_ and AVA also in modern CT scanners.

## Introduction

Assessment of left ventricular (LV) parameters, left ventricular outflow tract area (LVOT_area_) and aortic valve area (AVA) are common practice in echocardiography and cardiac magnetic resonance tomography (CMR).^1–3^ Echocardiography has a high temporal resolution but it is user-dependent and can be inconclusive due to poor acoustic windows. CMR is considered the gold-standard for LV assessment but is a costly and time-consuming examination with limited access.

Cardiac CT has been established for non-invasive coronary angiography and pre-interventional planning of structural heart interventions.^4,5^ Spiral acquisition during the entire cardiac cycle with retrospectively ECG-gated image reconstruction allows the assessment of functional LV parameters.^6^ CT provides high spatial and reasonable temporal resolution with current CT scanner systems. However, any more routine clinical use for LV, LVOT_area_ and AVA assessment would be restricted by the need of radiation and contrast. All the same, when echocardiography or CMR are contraindicated or inconclusive, CT might offer a reliable alternative for LV function, LVOT_area_ and aortic valve stenosis severity assessment. Recent studies showed a good agreement between LV parameters and AVA obtained by CT compared to CMR and echocardiography.^6–11^ However, there is only scarce data on the influence of CT reconstruction parameters on respective measurements. Furthermore, most previous studies have been performed with 16- or 64-slice CT scanner and their results do not represent the current state of the art in CT technology.^12–14^

The aim of the present study was to investigate the influence of the CT-reconstruction parameters slice thickness, number of phases of the cardiac cycle, matrix size and iterative reconstruction (IR) algorithm on the assessment of LV parameters, LVOT_area_ and AVA.

## Methods and materials

### Patients

Eighty patients who underwent contrast-enhanced cardiac CT for standard transcatheter aortic valve replacement work-up between December 2016 and April 2017 were screened for enrollment. After excluding patients with regional wall motion abnormalities and/or LV aneurysm based on prior echocardiography (31 patients) or an artificial aortic valve (four patients), the final cohort counted 45 patients. Covariates, including cardiac history and risk factors, were taken from the patient data records.

### CT data acquisition

A third-generation dual-source CT scanner (Somatom Definition Force, 250 ms gantry rotation, 66 ms temporal resolution, 2 × 196 × 0.6 collimation, Siemens Healthineers, Forchheim, Germany) with spiral acquisition and retrospectively ECG-gated image reconstruction was applied. Tube voltage and current were set at 100 kV/500 mAs in patients with 60–100 kg and weight-adapted in patients below or above this weight range. Contrast agent transit time was measured using test bolus technique. CT angiography was performed with 60-ml contrast agent (5 ml s^−1^), followed by a 50-ml flush consisting of 80% saline and 20% contrast agent using a dual-head power injector. Patients were not administered additional oral or intravenous beta-blockers for the scan according to our standard TAVI CT protocol.

### Image reconstruction

All datasets were reconstructed with a medium sharp convolution kernel (‘Bv40’). For the standard reconstruction, a filtered back-projection algorithm with 0.6-mm-slice thickness, 0.4-mm increment, 20 cardiac phases (5% steps throughout the entire R-R interval, 0–95%) and a 512 × 512 matrix was applied. Additional data sets were reconstructed with modifications in slice thickness (1, 2, 5 and 8 mm, increment 0.4 mm), number of cardiac phases [10 (10% steps, 0–90%) and 5 (20% steps, 0–80%)], matrix size (256 × 256) and an iterative reconstruction (IR) algorithm (ADMIRE, Siemens Healthineers, strength level 2 out of 5, number increases with the amount in noise reduction), but otherwise identical parameters to the standard reconstruction.

### Image analysis

**Figure 1.**
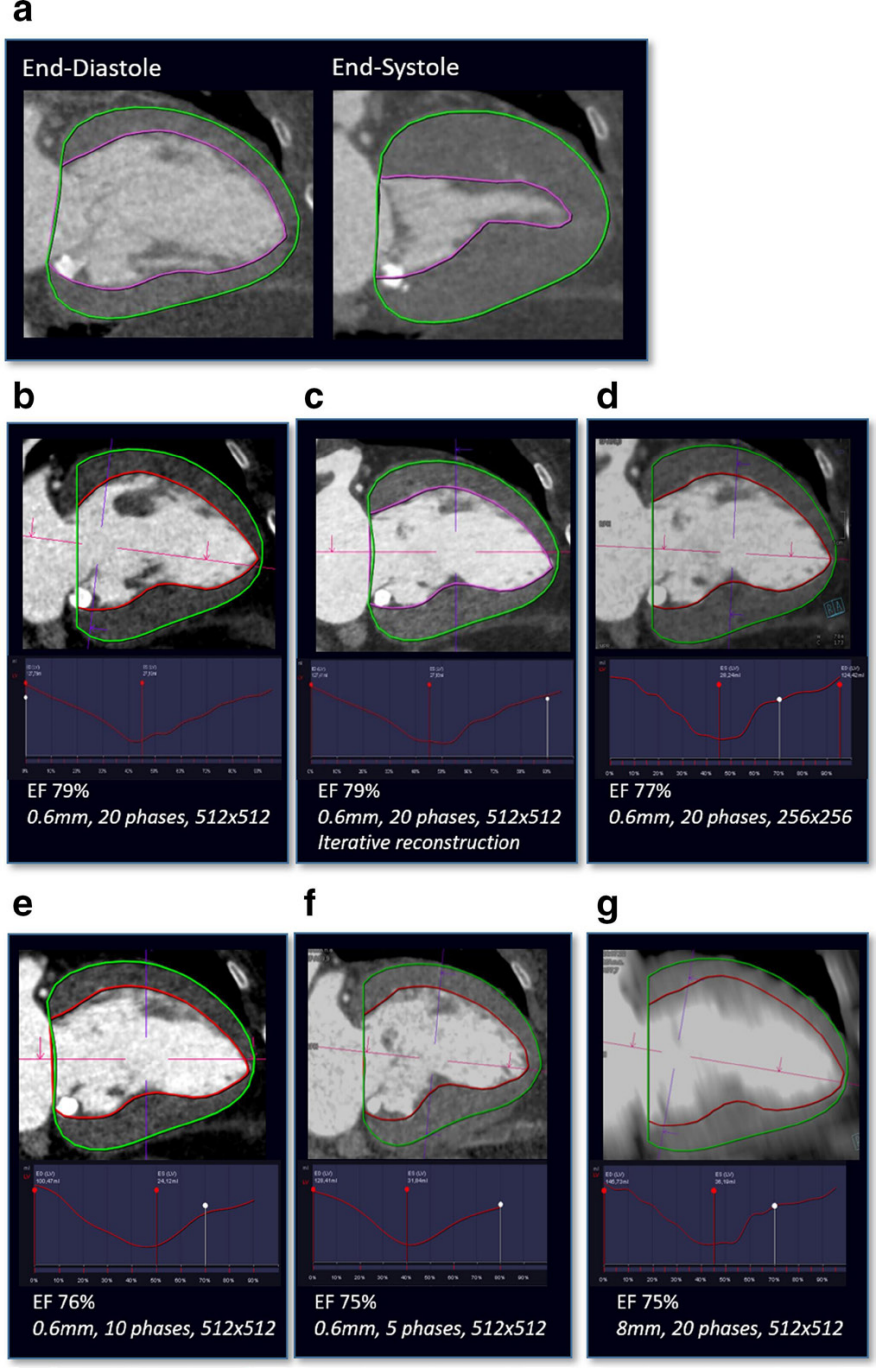
(a) Automatic diastolic and systolic contour for LV functional assessment. (**b–**g) Assessment of left ventricular ejection fraction in a (b) standard reconstruction with 0.6-mm slice thickness, 20 phases of the cardiac cycle and 512 × 512 matrix size and modified reconstructions with (c) an iterative reconstruction algorithm, (**D**) a reduction in matrix size to 256 × 256, a reduction in the number of reconstructed phases of the cardiac cycles to (e) 10 (10% steps between 0 and 90%) and (f) 5 (20% steps between 0 and 80%) and (g) an increase of slice thickness to 8 mm.

Post-processing was performed using Syngo.via cardiac function module (Siemens Healthineers). The reader was blinded to the reconstruction information. Datasets were visually assessed for diagnostic image quality using the 5-point Likert scale (1 = excellent, 5 = uninterpretable). Image quality parameters included homogeneity of contrast distribution and contrast between LV cavity and myocardium to allow automated segmentation. Automated tracing of the endocardial borders was visually verified by the reader and manually corrected, if necessary (Figure 1a). Papillary muscles and trabeculae were included in the ventricular lumen. After operator approval of the contours, LV ejection fraction (EF), stroke volume (SV), end-diastolic (EDV) and end-systolic volume (ESV) were automatically computed. A high accuracy for automated LV assessment by CT has been demonstrated.^15^ The selected phases of end-diastole and end-systole were recorded as percent R-R-interval values for each dataset.

**Figure 2.**
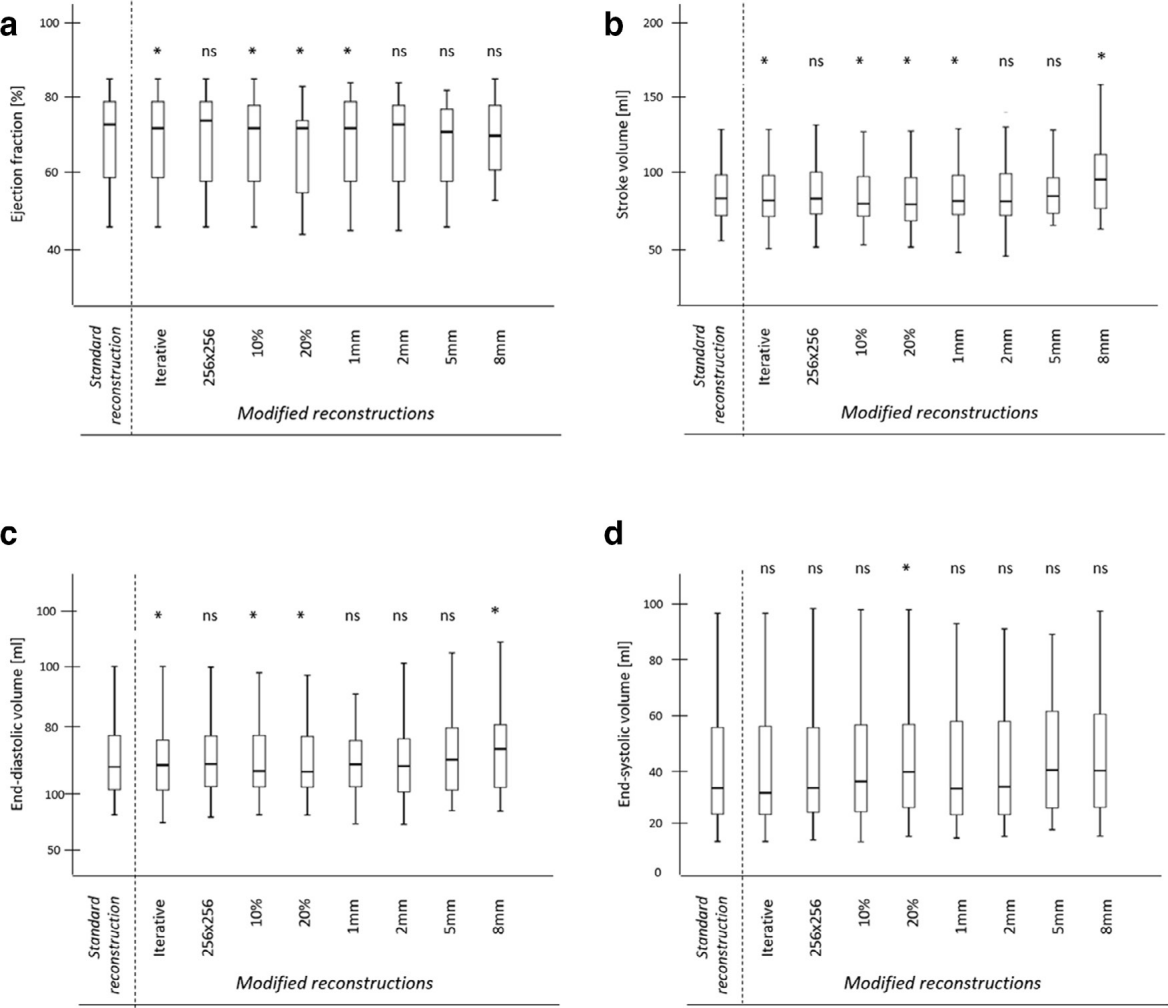
Box-plot diagrams for (a) left ventricular ejection fraction, (b) stroke volume, (c) end-diastolic and (d) end-systolic volume. Median, interquartile range and maximum and minimum values are presented. *, *p* < 0.05; ns, non-significant.

For assessment of the LVOT_area_ and AVA, a multiplanar image display mode was used with reference images in oblique coronal and sagittal planes along the LVOT. Measurements of the AVA were performed in a double-oblique transverse plane across the aortic valve at the level where the orifice was smallest and of the LVOT just below the aortic valve plane, as previously described.^11,16^ Determination of the anatomic AVA was then performed by manual planimetry at the systolic phase with the widest valve opening by tracing the orifice along the edges of the cusps. LVOT_area_ was assessed in the same phase of the cardiac cycle. High intra- and/or interobserver variability for this approach has been demonstrated in prior studies.^11,16^

### Statistics

Statistical analysis was performed using SPSS software (IBM^®^ SPSS^®^ statistics, version 19 for Windows). Continuous variables were expressed as mean ± SD or median, categorical variables as frequencies and percentage, unless otherwise specified. Correlation was assessed using Pearson’s test. Statistical significance between the standard reconstruction and the modified reconstructions was assessed using the paired t-test for normally distributed data or the paired Wilcoxon test for non-normally distributed data. The 95% limits of agreement were defined using Bland-Altman analysis. *p* < 0.05 was considered to be statistically significant.

## Results

Mean age was 79 ± 6 years, 62% were male (Table 1). The mean BMI was 26.7 ± 5.6 kg/m². The average heart rate was 65 ± 11 beats/min (range 39–89 beats/min), 18 patients were in atrial fibrillation (40%). Tube voltage was set at 90 kV in one patient (2%), 100 kV in 42 patients (94%) and 110 kV in two patients (4%). Image quality was rated as excellent in 33 (73%), good in 9 (20%) and acceptable in 3 (7%) patients. For an exemplary data set, we present time for reconstruction and storage volume for each reconstruction in Supplementary Table 1.

Supplementary Table 1.

**Table 1.** Baseline demographics and acquisition parameters

*n* = **45**	Value
Patient characteristics	
Age (y)	79 ± 6
Male, n (%)	28 (62)
BMI (kg/m^2^)	26.7 ± 5.6
Body weight (kg)	75.9 ± 16.7
Medication, n (%)	
Beta-blockers	26 (58)
Ivabridine	0 (0)
CT parameters	
Tube voltage, n (%)	
90 kV	1 (2)
100 kV	42 (94)
110 kV	2 (4)
Rhythm during acquisition, n (%)	
Sinus rhythm	27 (60)
Atrial fibrillation	18 (40)
Acquisition heart rate (beats/min)	65 ± 11 (range 39–89)
Radiation exposure parameters	
Dose-length product (mGy*cm)	1230 ± 222 (median 1190, range 898–1740)
Effective dose (mSv)	1.7 ± 0.4
Time to peak bolus (s)	20.4 ± 1.5 (range 18–24)

### Left ventricular function parameters

**Figure 3.**
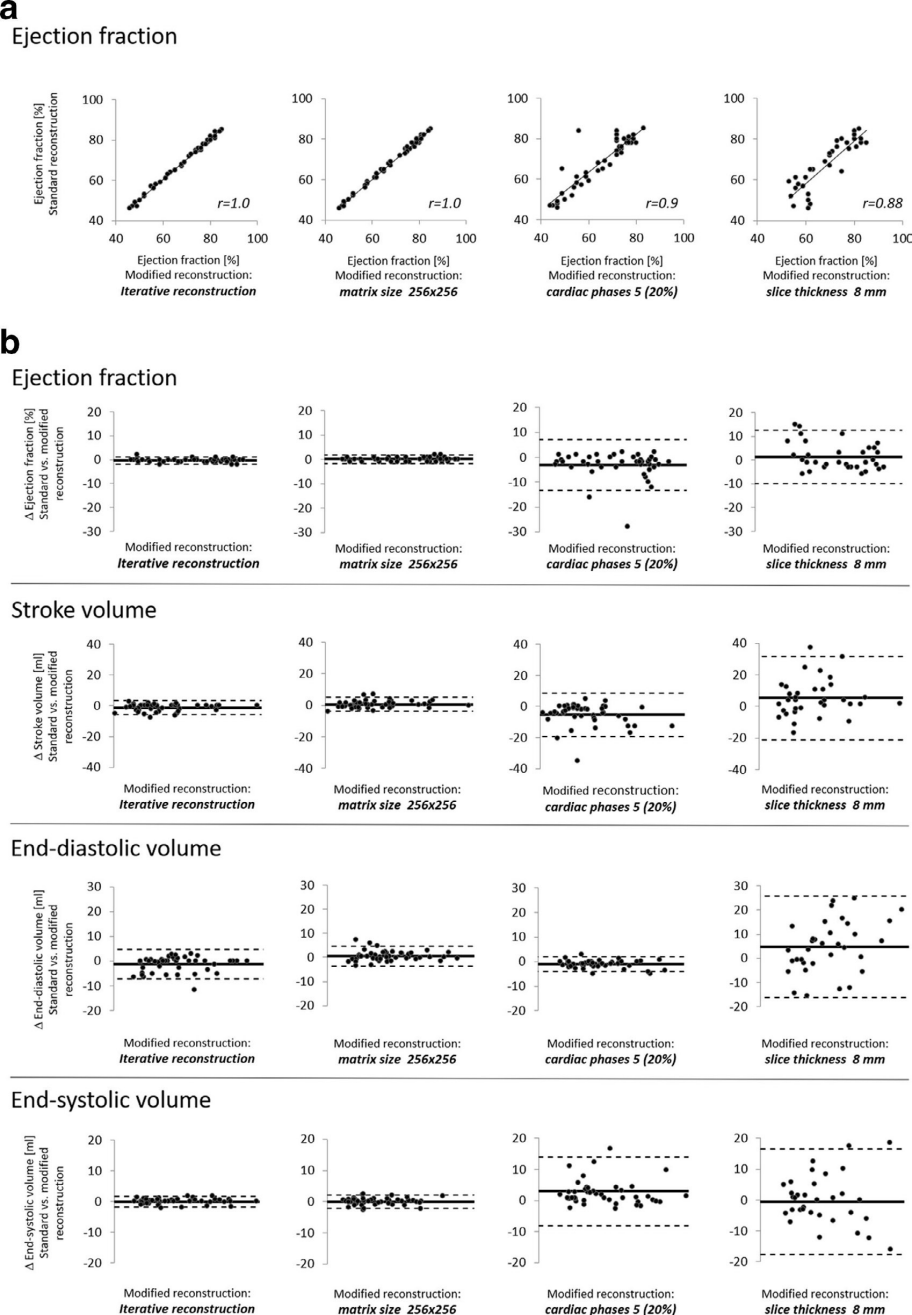
(a) Correlation graph for left ventricular ejection fraction and (b) Bland-Altman analysis for left ventricular ejection fraction, stroke volume, end-diastolic and end-systolic volume for modified reconstructions with an iterative reconstruction, a reduction in matrix size to 256 × 256, a reduction of the phases of the cardiac cycle to 5 (20% steps between 0–80%) and an increase in slice thickness of 8 mm in comparison with the standard reconstruction.

On standard reconstructed data sets, mean EF was 69±12%, SV 87.6 ± 21.9 ml, EDV 129.5 ± 31.1 ml and ESV 41.9 ± 21.8 ml (Table 2, Figures 1b–g and 2). Pearson’s correlation coefficient was overall high in all modified reconstructions compared to standard reconstruction (Figure 3). The lowest correlation with the standard data set was observed in 8-mm slice thickness reconstructions (r-value: EF = 0.88; SV = 0.87; EDV = 0.94; ESV = 0.92, all *p* < 0.001). There was no significant difference in LV parameters when the matrix size was decreased from 512 × 512 to 256 × 256. The use of IR algorithm level 2 compared to standard reconstruction with filtered-back-projection resulted in a minor, but statistically significant underestimation in EF, SV and EDV, whereas the ESV was not significantly changed. A similar effect was observed for an increased slice thickness of 1 mm with a minor, but statistically significant underestimation in EF and SV. A stronger increase in slice thickness resulted in a higher scattering of the LV parameters compared to the standard reconstruction. Here, 8-mm slice thickness reconstruction resulted in a statistically significant overestimation of SV (*p* = 0.015) and EDV (*p* = 0.016) compared to the standard reconstruction. In contrast, reducing the number of reconstructed phases of the cardiac cycle from 20 to 10 and 5 resulted in a statistically significant overestimation in ESV (both *p* < 0.001) and an underestimation in EF, SV and EDV (all *p* < 0.001) compared to the standard reconstruction. The mean end-systolic phase was 38±5% for the standard reconstruction and showed a statistically significant difference only for the modified reconstruction reduced to five phases of the cardiac cycle (39±4%, *p* = 0.032).

**Table 2.** Assessment of global left ventricular ejection fraction using standard reconstruction and modified reconstructions

Standard reconstruction		Modified reconstructions							
	0.6 mm 5% 512 × 512 FBP	0.6 mm 5% 512 × 512 IR2	0.6 mm 5% 256 × 256 FBP	0.6 mm 10% 512 × 512 FBP	0.6 mm 20% 512 × 512 FBP	1 mm 5% 512 × 512 FBP	2 mm 5% 512 × 512 FBP	5 mm 5% 512 × 512 FBP	8 mm 5% 512 × 512 FBP
Ejection fraction									
Mean ± SD (%)	69 ± 12	68 ± 12	69 ± 12	67 ± 12	66 ± 12	68 ± 12	68 ± 12	68 ± 11	69 ± 10
Median (%)	73	72	74	72	72	72	73	71	70
*Pearson Correlation compared to standard reconstruction*									
Coefficient		1.0	1.0	0.99	0.90	0.99	0.98	0.97	0.88
*p*-value		<0.001	<0.001	<0.001	<0.001	<0.001	<0.001	<0.001	<0.001
*Bland-Altman Analysis compared to standard reconstruction*									
95% limits of agreement		1.3; −1.8	1.8;−1.6	1.7;−4.1	7.0; −13.3	2.4;−3.6	3.7;−4.5	6.9;−7.4	12.5;−9.8
Mean bias		−0.30	0.1	−1.2	−3.1	−0.6	−0.4	−0.2	1.3
*p* value		0.022	0.594	<0.001	<0.001	0.011	0.332	0.577	0.436
Stroke Volume									
Mean ± SD (ml)	87.6 ± 21.9	86.4 ± 22.1	88.1 ± 22.3	85.0 ± 20.8	82.3 ± 20.7	85.4 ± 20.3	86.0 ± 20.4	88.4 ± 21.0	94.5 ± 23.4
Median (ml)	82.2	80.8	82.1	78.6	78.2	80.5	80.3	83.7	94.8
*Pearson Correlation compared to standard reconstruction*									
Coefficient	0.99	0.99	0.99	0.95	0.98	0.96	0.91	0.87	
*p* value	<0.001	<0.001	<0.001	<0.001	<0.001	<0.001	<0.001	<0.001	
*Bland-Altman Analysis compared to standard reconstruction*									
95% limits of agreement	3.3; −5.7	5.0;−4.0	3.7;−8.9	8.7; −19.3	7.4;−11.8	10.6;−13.9	14.5;−21.2	31.8;−21.1	
Mean bias		−1.2	0.5	−2.6	−5.3	−2.2	−1.6	−0.8	5.3
*p* value		0.003	0.218	<0.001	<0.001	0.021	0.307	0.939	0.015
End-diastolic volume									
Mean ± SD (ml)	129.5 ± 31.1	128.2 ± 31.5	130.0 ± 31.1	127.9 ± 30.4	127.4 ± 30.4	127.6 ± 29.4	127.9 ± 29.4	132.9 ± 31.3	138.2 ± 35.3
Median (ml)	124.4	125.9	126.8	120.9	120.5	126.5	125.1	130.4	139.2
*Pearson Correlation compared to standard reconstruction*									
Coefficient	1.0	1.0	1.0	1.0	0.99	0.97	0.96	0.94	
*p* value	<0.001	<0.001	<0.001	<0.001	<0.001	<0.001	<0.001	<0.001	
*Bland-Altman Analysis compared to standard reconstruction*									
95% limits of agreement	4.7; −7.3	4.7;−3.5	3.4;−6.5	2.0; −4.1	8.9;−12.7	12.3;−15.4	20.5;−21.5	25.7;−16.2	
Mean bias		−1.3	0.6	−1.6	−1.1	−1.9	−1.6	−0.5	4.7
*p* value		0.026	0.184	<0.001	<0.001	0.160	0.786	0.411	0.016
End-systolic volume									
Mean ± SD (ml)	41.9 ± 21.8	41.9 ± 21.9	41.9 ± 21.9	42.9 ± 21.7	44.8 ± 21.2	42.2 ± 21.7	41.9 ± 21.1	44.5 ± 20.9	43.7 ± 20.9
Median (ml)	33.4	31.6	33.4	35.8	39.4	33.2	33.8	40.0	39.8
Pearson Correlation compared to standard reconstruction									
Coefficient		1.0	1.0	1.0	0.97	0.99	1.0	40.0	39.8
p-value		<0.001	<0.001	<0.001	<0.001	<0.001	<0.001	<0.001	<0.001
Bland-Altman Analysis compared to standard reconstruction									
95% limits of agreement		1.8; −1.7	2.2; −2.1	4.3; −2.2	13.9; −8.0	3.7; −3.1	5.2; −5.2	10.6; −10.1	16.5; −17.6
Mean bias		0	0	1.0	2.9	0.3	0	0.2	−0.6
*p* value		0.742	0.678	<0.001	<0.001	0.146	0.536	0.878	0.617
End-systolic phase for measurement									
Mean ± SD (%)	38 ± 5	37 ± 5	37 ± 5	37 ± 6	39 ± 4	37 ± 5	38 ± 10	38 ± 5	38 ± 5
*p* value		0.414	0.102	0.162	0.032	0.655	0.782	0.102	0.331

IR, iterative reconstruction; FBP, filtered back projection.

When comparing patients with sinus rhythm to those with atrial fibrillation, a minor underestimation in EF, SV and EDV for IR algorithm level two compared to standard reconstruction with filtered-back-projection was observed in patients with sinus rhythm but not in atrial fibrillation (Supplementary Table 2). Otherwise, the heart rhythm did not affect the assessment of LV parameters in standard compared to modified reconstructions.

Supplementary Table 2.

### Left ventricular outflow tract assessment

**Figure 4.**
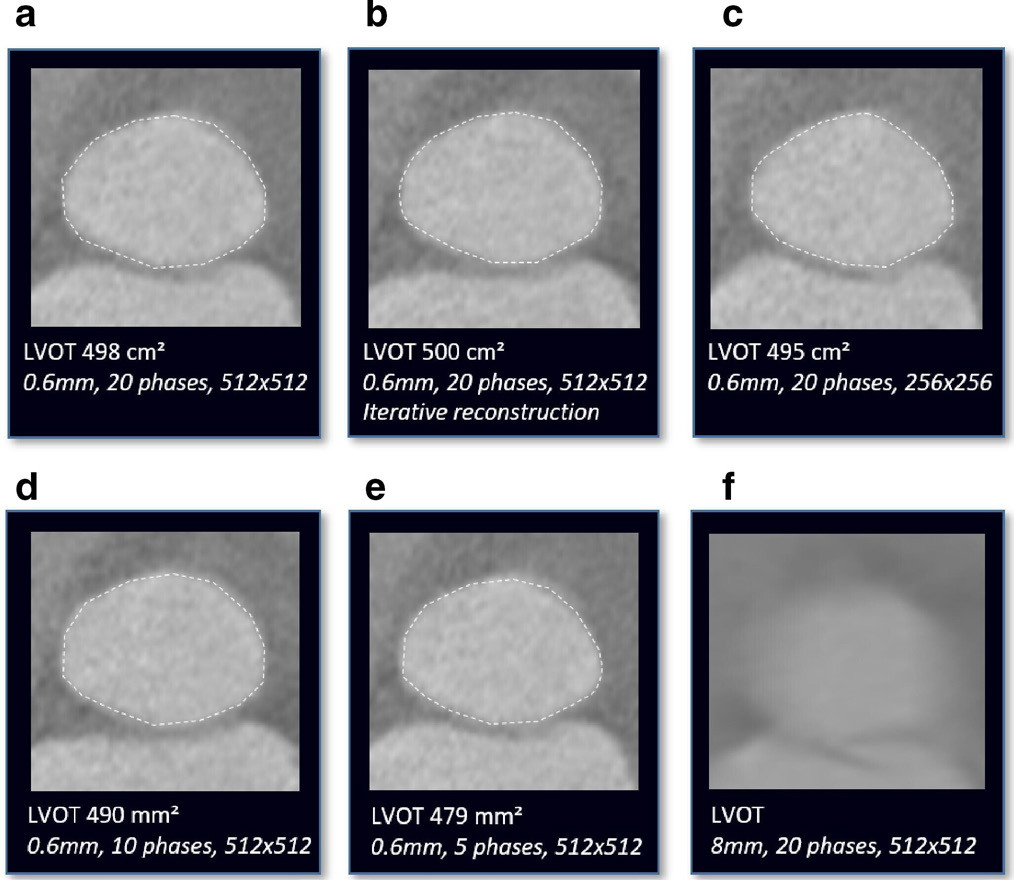
Manual assessment of LVOT area in a (a) standard reconstruction with 0.6-mm slice thickness, 20 phases of the cardiac cycle and 512 × 512 matrix size and modified reconstructions with (b) an iterative reconstruction algorithm, (c) a reduction in matrix size to 256 × 256, a reduction in the number of reconstructed phases of the cardiac cycles to (d) 10 (10% steps between 0 and 90%) and (e) 5 (20% steps between 0 and 80%). (f) An increase of slice thickness to 8 mm is not suitable for LVOT assessment due to an inaccurate outline.

**Figure 5.**
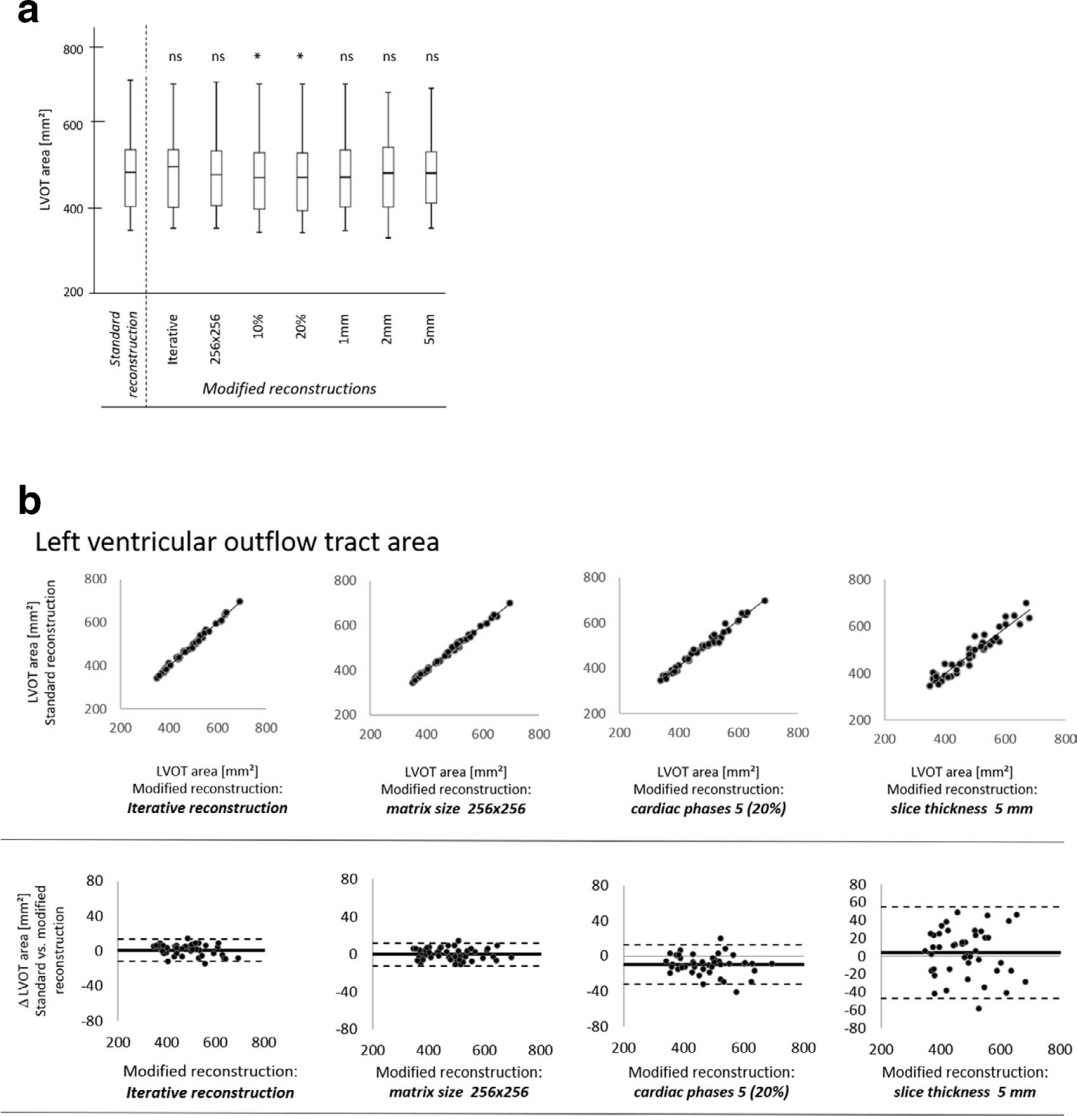
(a) Box-plot diagrams for LVOT area. Median, interquartile range and maximum and minimum values are presented. *, *p* < 0.05; ns, non-significant. (b) Bland-Altman analysis with corresponding correlation graph of LVOT area assessment for modified reconstructions with an iterative reconstruction, a reduction in matrix size to 256 × 256, a reduction of the phases of the cardiac cycle to 5 (20% steps between 0 and 80%) and an increase in slice thickness to 5 mm comparison with standard reconstruction.

In the standard reconstructed data set, the mean LVOT_area_ was 482 ± 90 mm² (Table 3, Figures 4 and 5). In reconstructions with 8-mm slice thickness, the LVOT outline could overall not be clearly defined from the surrounding tissue. In the remaining modified reconstructions, the lowest correlation for LVOT_area_ with the standard reconstruction was found for reconstructions with 5-mm slice thickness (*r* = 0.96, *p* < 0.001). There was no significant difference in LVOT_area_ for reconstructions with a modified matrix size, IR and slice thickness compared to the standard reconstruction. A statistically significant underestimation for LVOT_area_ was only seen in reconstructions with 10 and 5 phases of the cardiac cycle (both *p* < 0.001). The heart rhythm (sinus rhythm *vs* atrial fibrillation) did not have any significant effect on LVOT measurement between standard and modified reconstructions.

**Table 3.** Assessment of left ventricular outflow tract area using standard reconstruction and modified reconstructions

Standard reconstruction		Modified reconstructions						
	0.6 mm 5% 512 × 512 FBP	0.6 mm 5% 512 × 512 IR2	0.6 mm 5% 256 × 256 FBP	0.6 mm 10% 512 × 512 FBP	0.6 mm 20% 512 × 512 FBP	1 mm 5% 512 × 512 FBP	2 mm 5% 512 × 512 FBP	5 mm 5% 512 × 512 FBP
Mean ± SD (mm²)	482 ± 90	482 ± 88	481 ± 90	477 ± 89	472 ± 88	481 ± 89	483 ± 89	486 ± 89
Median (mm²)	482	495	476	469	460	470	480	480
*Pearson Correlation compared to standard reconstruction*								
Coefficient		1.0	1.0	0.99	0.99	0.99	0.98	0.96
*p* value		<0.001	<0.001	<0.001	<0.001	<0.001	<0.001	<0.001
*Bland-Altman Analysis compared to standard reconstruction*								
95% limits of agreement		13.2; −11.9	11.8;−12.4	11.5;−21.6	12.6; −32.4	22.8;−24.4	39.9;−38.3	54.6;−46.9
Mean bias		0.6	−0.3	−5.0	−9.9	−0.8	−0.4	3.9
*p* value		0.427	0.676	<0.001	<0.001	0.553	0.933	0.262

FBP, filtered back projection;IR, iterative reconstruction.

### Aortic valve orifice area

**Figure 6.**
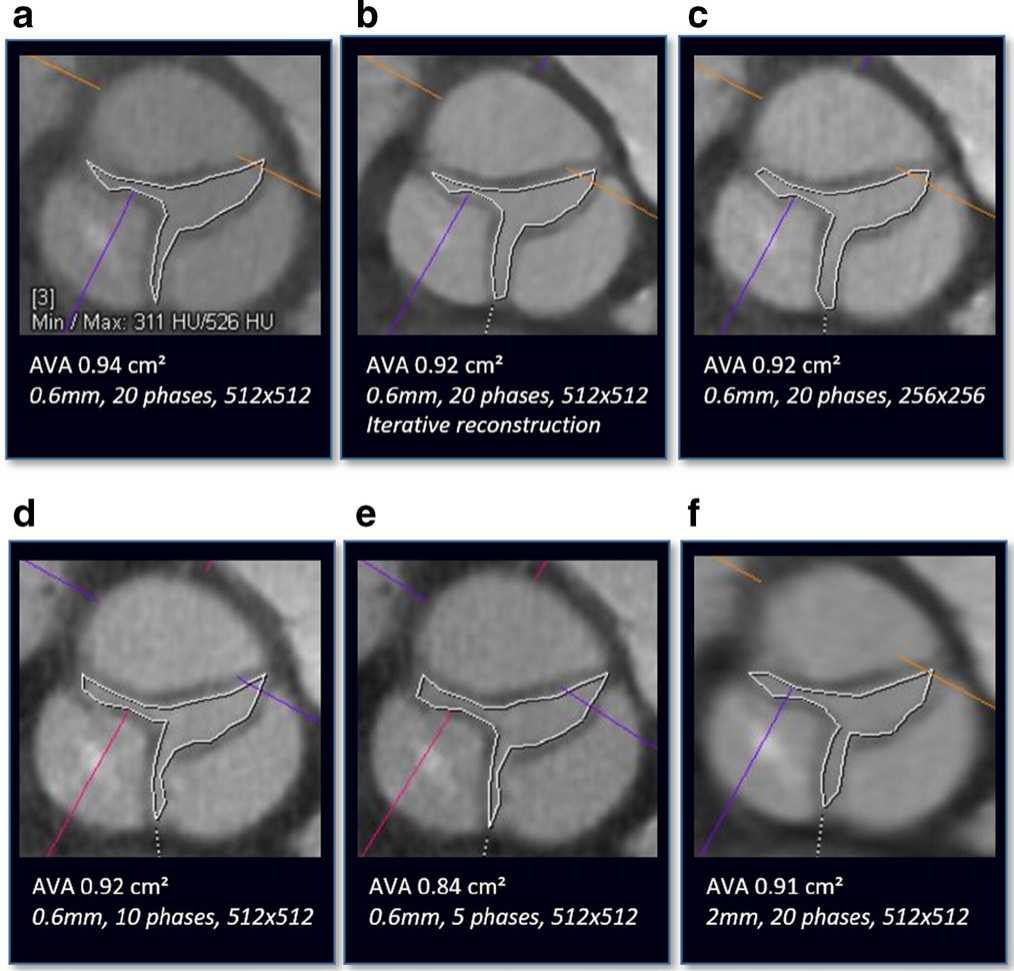
Manual assessment of AVA in a (a) standard reconstruction with 0.6 mm slice thickness, 20 phases of the cardiac cycle and 512 × 512 matrix size and modified reconstructions with (b an iterative reconstruction algorithm, (c) a reduction in matrix size to 256 × 256, a reduction in the number of reconstructed phases of the cardiac cycles to (d) 10 (10% steps between 0 and 90%) and (e) 5 (20% steps between 0 and 80%) and (f) an increase of slice thickness to 2 mm.

**Figure 7.**
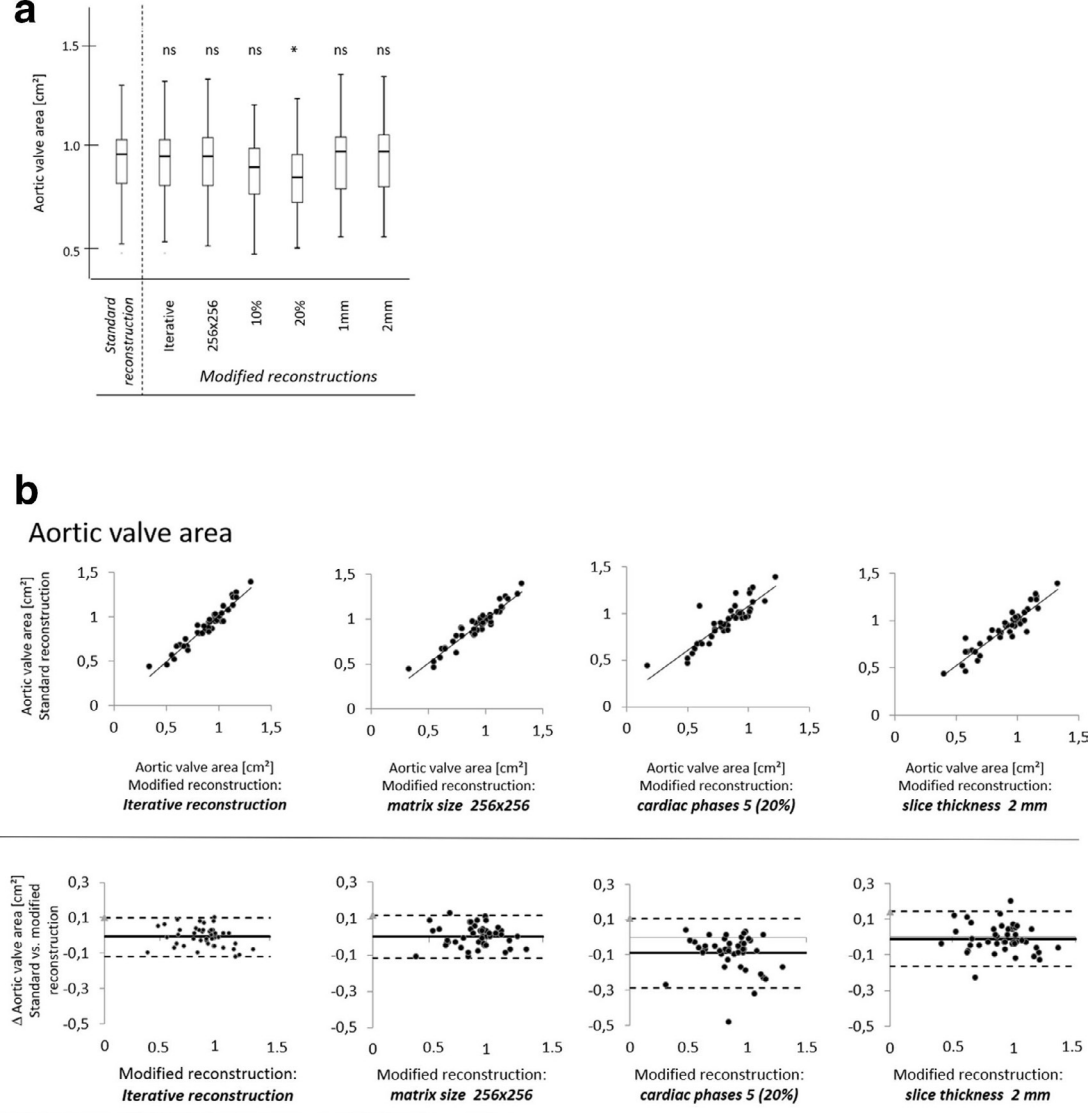
(a) Box-plot diagrams for AVA. Median, interquartile range and maximum and minimum values are presented. *, *p* < 0.05; ns, non-significant. (b) Bland-Altman analysis with corresponding correlation graph of AVA assessment for modified reconstructions with an iterative reconstruction, a reduction in matrix size to 256 × 256, a reduction of the phases of the cardiac cycle to 5 (20% steps between 0 and 80%) and an increase in slice thickness to 2 mm comparison to standard reconstruction.

In the standard reconstructed data set, the mean AVA was 0.91 ± 0.21 cm² (Table 4, Figures 6 and 7). In reconstructions with ≥5 mm slice thickness, an accurate planimetry of the AVA was not feasible in 56% of patients due to an indistinct orifice border outline. For all other modified reconstructions, the lowest correlation with the standard reconstruction was found for reconstructions with only five phases of the cardiac cycle (*r* = 0.88, *p* < 0.001). There was no significant difference in AVA for reconstructions with modified matrix size, IR and 1- and 2-mm slice thickness compared to the standard reconstruction. A statistically significant underestimation for AVA was only seen in reconstructions with 10 and 5 phases of the cardiac cycle (both *p* < 0.001). The mean phase of the cardiac cycle for AVA determination was 14±4% in the standard reconstruction. Except for the modified reconstruction with five phases (all measurements at 20% of the cardiac cycle), there was no significant difference in the mean phase of AVA measurement for the other modified reconstructions compared to the standard reconstruction. The heart rhythm (sinus rhythm *vs* atrial fibrillation) did not have any significant effect on AVA measurement between standard and modified reconstructions.

**Table 4.** Assessment of aortic valve area using standard reconstruction and modified reconstructions

Standard reconstruction		Modified reconstructions					
	0.6 mm 5% 512 × 512 FBP	0.6 mm 5% 512 × 512 IR	0.6 mm 5% 256 × 256 FBP	0.6 mm 10% 512 × 512 FBP	0.6 mm 20% 512 × 512 FBP	1 mm 5% 512 × 512 FBP	2 mm 5% 512 × 512 FBP
Aortic valve area							
Mean ± SD (cm²)	0.91 ± 0.21	0.90 ± 0.20	0.91 ± 0.21	0.88 ± 0.21	0.82 ± 0.20	0.90 ± 0.21	0.90 ± 0.20
Median (cm²)	0.95	0.94	0.96	0.89	0.84	0.94	0.96
*Pearson Correlation compared to standard reconstruction*							
Coefficient		0.97	0.96	0.97	0.88	0.96	0.93
*p* value		<0.001	<0.001	<0.001	<0.001	<0.001	<0.001
*Bland-Altman Analysis compared to standard reconstruction*							
95% limits of agreement		0.10; −0.12	0.12;−0.12	0.07;−0.14	0.11; −0.29	0.10; −0.12	0.14;−0.16
Mean bias		−0.01	0.0	−0.04	−0.09	−0.01	−0.01
*p* value		0.354	0.938	<0.001	<0.001	0.120	0.341
*Bland-Altman Analysis compared to standard reconstruction*							
End-systolic phase for measurement							
Mean ± SD (%)	14 ± 4	14 ± 4	14 ± 4	15 ± 5	20 ± 0	14 ± 5	14 ± 4
*p* value		0.290	0.617	0.269	<0.001	0.796	0.617

FBP, filtered back projection; IR, iterative reconstruction.

## Discussion

The present study systematically investigated the influence of reconstruction parameters on the assessment of left ventricular function, LVOT and aortic valve area. To our knowledge, this study provides the most comprehensive investigation in terms of the variety of assessed reconstruction parameters including slice thickness, number of phases of the cardiac cycle, matrix size and IR algorithm. Our study revealed that reconstructions with a decrease in the number of phases of the cardiac cycle resulted in a statistically significant underestimation in EF, SV, EDV, LVOT_area_ and perimeter and AVA and overestimation in ESV compared to the standard reconstruction. There was a minor difference for reconstructions with an applied IR algorithm and 1-mm slice thickness. Overall, an increase in slice thickness showed a higher scattering of the measurements.

CT offers a reliable alternative for assessment of LV parameters, LVOT_area_ and AVA in patients with poor image windows and contraindications for echocardiography and CMR. A high temporal and spatial resolution are crucial for exact LV assessment, LVOT_area_ and AVA. Several factors affect CT spatial resolution including, for example, pixel size, field of view, slice thickness, detector size, reconstruction algorithm and also patient-dependent factors like motion.^17^ Temporal resolution, on the other side, can be influenced by, for example, gantry rotation time, acquisition mode, type of image reconstruction and pitch.^17^ Whereas CT offers an excellent spatial resolution, the temporal resolution is only moderate compared to echocardiography and CMR. One could assume that the more precise and detailed the CT reconstruction parameters are chosen, the more accurate the assessment should be. On the other hand, the more detailed the reconstruction is, the longer it takes for image reconstruction, data post-processing and LV parameter analysis and also the larger the amount of data storage. There are only few studies evaluating the influence on modified CT reconstruction parameters on LV function.^12–14^ Ko et al^12^ found no significant difference in LV function assessment between 20- and 10-phase reconstructions. Suzuki et al^13^ reported an underestimation of EF when only three instead of six systolic cardiac phases were considered. Both studies were performed with 16- or 64-slice CT scanners which achieve a substantially lower temporal resolution than current dual source CT systems. Although a high temporal resolution is crucial for the selection of the exact end-diastolic and -systolic phase and thereby for calculating accurate LV parameters. To capture the correct end-systolic phase, a temporal resolution of ~50 ms is required as demonstrated for other image modalities. 64-slice scanners offer only a temporal resolution between 125 and 250 ms, whereas dual source CT offers a temporal resolution of up to 66 ms.^18^ Furthermore, the variability of temporal resolution according to the heart rate has been shown to limit a reliable evaluation of LV function in CT scanners with 64 slices or less.^12^ In contrast, dual source CT provides high diagnostic image quality over a wide range of heart rates with a heart rate-independent temporal resolution.^19^ For a dual-source CT-based approach, we found a statistically significant underestimation in EF, SV and EDV and overestimation in ESV when reducing the number of reconstructed phases from 20 to 10 and even more for a reduction to five phases. We assume that this observed difference was due to a more precise selection of the correct end-systolic phase in data sets with more approximate reconstructions.

Slice thickness determines the through-plane (or z-axis) spatial resolution. The larger the slice thickness, the lower the resolution. Suzuki et al^13^ reported no significant difference in EF calculated from end-systolic images with 1, 2 and 3 mm slice thickness. Vural et al^14^ demonstrated no significant difference in EDV calculated from 1- and 2-mm-reconstructions but reported minor but statistically significant differences between reconstructions in EF and ESV. In our present study, we also observed very minor, but statistically significant differences for EF and SV between 0.6 and 1 mm slice thickness which we do not see as clinically relevant. Further increases in slice thickness (2, 5 and 8 mm), although, showed a larger scattering of the measurements which is most-likely due to the decrease in spatial resolution and, thereby, a more inaccurate distinction between endocardial tissue and ventricular lumen. In reconstructions with 8-mm slice thickness, SV and EDV were statistically significantly overestimated.

The matrix size determines the axial pixel size which is the ratio of field of view to image matrix.^20^ Pixel size is inversely related to in-plane spatial resolution. A reduction of the matrix size from 512 × 512 to 256 × 256 results in an increase in pixel size and, thereby, in a reduction in in-plane spatial resolution. We observed no significant difference in LV parameters derived from a conventional 512 × 512 compared to a 256 × 256 matrix.

Filtered-back-projection is based on several assumptions that simplify CT geometry as a compromise between reconstruction speed and image noise.^21^ Advances in computer processing power have made IR algorithms clinically feasible, resulting in reconstructed data with reduced image noise within an acceptable time.^22^ IR has been shown to potentially alter spatial resolution and image texture.^21^ Here, we used an IR strength level 2 out of 5. When comparing IR images with filtered-back-projection and otherwise non-modified reconstruction parameters, there was a minor, but statically significant underestimation in EF, SV and EDV, which will be most likely due to a smoothing of the image.

CT-based assessment of the LVOT has been shown to be superior to 2D echocardiography given its oval rather than circular shape. Reliable measurements of the LVOT are crucial for aortic valve stenosis and LVOT obstruction assessment. Halpern et al^16^ demonstrated that the LVOT area is slightly larger with a more circular shape in systole than in diastole. In the present study, we found a small but significant underestimation in LVOT area for 10 and 20% reconstruction steps compared to the standard reconstruction, which is likely caused by the restriction in the optimal systolic phase available for assessment. Reconstructions with a slice thickness of 8 mm are not suitable for LVOT assessment due to an inaccurate outline. An increase of up to 5 mm, a reduction in matrix size or use of IR showed no statistically significant difference in LVOT_area_.

To our knowledge, this study is the first to assess the influence of modified reconstruction parameters on CT-based assessment of aortic valve area. Prior studies have shown that planimetric AVA measurements on multidector-CT allow accurate grading of aortic valve stenosis severity.^11^ We noticed a statistically significant underestimation in AVA when only 10 or 5 phases of the cardiac cycle were reconstructed compared to standard reconstructed data. This is most likely due to the limited choice of phases to select the optimal systolic AVA. Whereas in standard reconstructed data the mean phase of the cardiac cycle for AVA determination was 13.8±4.2%, the AVA planimetry was performed at 20% of the cardiac cycle in all patients when the phases of the cardiac cycle were reduced to 5. With an increase of ≥5 mm slice thickness, AVA could not be longer accurately distinguished from the valve leaflets in more than half of the patients. A minor increase of up to 2 mm, a reduction in matrix size or use of IR showed no statistically significant difference in AVA.

Finally, the important question arises of the clinical relevance of the described findings. We believe that the reported minor differences for modified reconstructions with an increase in slice thickness ≤2 mm, a 256 × 256 matrix size and IR algorithm do not influence the clinical decision-making. However, if the slice thickness is increased ≥5 mm or the number of cardiac phases reduced, this may have a clinical relevance. Current recommendations for CT imaging in the context of transcatheter aortic valve implantation recommend reconstructions with <1 mm slice thickness,≤10% intervals of the cardiac cycle, a 512 × 512 matrix and filtered back projection or iterative reconstruction.^5^ Although we observed a small but significant difference in parameters if a 10% interval of the cardiac cycle compared 5% interval was used. The recommended parameters are standard for modern CT scanner generations. However, with the increased use of cardiac CT in daily routine, some hospital may use also earlier scanner generations and our current study points out the limitations in LV and AVA assessment if less accurate reconstruction parameters are applied.

### Limitations

We did not apply a gold standard like echocardiography or CMR for comparison but used the standard CT reconstruction applied in the latest-generation of dual source CT scanner. This was considered sufficient as agreement of LV function assessed by cardiac CT with echocardiography or CMR has been extensively investigated in earlier studies.^6–8^ To minimize confounders in the present study, assessment was not performed in patients with regional wall abnormalities and/or aneurysm of the left ventricle, as these patients were excluded. Accordingly, the mean ejection fraction was high compared to a real-life TAVI cohort. The number of patients included for analysis was only modest. We did not perform a dedicated inter- and intrareader analysis as a high accuracy for automated LV assessment by CT and a high intra- and/or interobserver variability for LVOT_area_ and AVA assessment has been demonstrated in earlier studies.^11,15,16^

## Conclusions

Cardiac CT is a robust method to determine LV function, LVOT_area_ and AVA. A marked reduction in the number of reconstructions per cardiac cycle as well as a substantial increase in slice thickness compared to standard reconstruction settings significantly affect the assessment of LV parameters, LVOT_area_ and AVA.
