# Supplementary material for: Analysis of left ventricular function, left ventricular outflow tract and aortic valve area using computed tomography: Influence of reconstruction parameters on measurement accuracy
Source: Br J Radiol. 2021 Jul 8;94(1124):20201306. doi: 10.1259/bjr.20201306 (PMC8523190; doi:10.1259/bjr.20201306)
Supplement: Supplementary Table 1. [file bjr.20201306.suppl-01.docx]

**Supplementary Table S1 - Time for reconstruction and storage volume** for an exemplary data set

| **Reconstructions** | | | | **No. of slices** | **Storage Volume [MB]** | **Time for reconstruction [seconds]** |
| --- | --- | --- | --- | --- | --- | --- |
| **Slice thickness** | **steps of cardiac phases** | **matrix size** | **algorithm** |  |  |  |
| 0.6 mm | 5% | 512x512 | FBP | 3680 | 2120 | 100 |
| 0.6 mm | 5% | 512x512 | IR2 | 3680 | 2120 | 101 |
| 0.6 mm | 5% | 256x256 | FBP | 3860 | 570 | 72 |
| 0.6 mm | 10% | 512x512 | FBP | 1737 | 1060 | 45 |
| 0.6 mm | 20% | 512x512 | FBP | 965 | 543 | 28 |
| 1 mm | 5% | 512x512 | FBP | 2320 | 1270 | 93 |
| 2 mm | 5% | 512x512 | FBP | 1160 | 660 | 89 |
| 5 mm | 5% | 512x512 | FBP | 480 | 264 | 61 |
| 8 mm | 5% | 512x512 | FBP | 300 | 171 | 59 |

MB, megabyte; FBP, filtered-back-projection algorithm; IR, iterative reconstruction.
