# Supplementary material for: Analysis of left ventricular function, left ventricular outflow tract and aortic valve area using computed tomography: Influence of reconstruction parameters on measurement accuracy
Source: Br J Radiol. 2021 Jul 8;94(1124):20201306. doi: 10.1259/bjr.20201306 (PMC8523190; doi:10.1259/bjr.20201306)
Supplement: Supplementary Table 2. [file bjr.20201306.suppl-02.docx]

**Table S2 – Comparison LV parameters and AVA between standard and modified reconstructions between patients in sinus rhythm and atrial fibrillation**

**Patients in Sinus rhythm (n=27)**

| Standard reconstruction | | | Modified reconstructions | | | | | | | |
| --- | --- | --- | --- | --- | --- | --- | --- | --- | --- | --- |
|  | 0.6mm  5%  512x512  FBP | | 0.6 mm  5%  512x512  IR2 | 0.6 mm  5%  256x256  FBP | 0.6 mm  10%  512x512  FBP | 0.6 mm  20%  512x512  FBP | 1 mm  5%  512x512  FBP | 2 mm  5%  512x512  FBP | 5 mm  5%  512x512  FBP | 8 mm  5%  512x512  FBP |
| **Ejection fraction** | | | | | | | | | | |
| Mean ± SD [%] | 72±11 | | 72±11 | 72±11 | 71±11 | 69±10 | 71±11 | 72±10 | 69±10 | 70±11 |
| Median [%] | 76 | | 76 | 77 | 76 | 72 | 75 | 76 | 73 | 73 |
| p-value | |  | 0.038 | 0.216 | <0.001 | <0.001 | 0.068 | 0.774 | 0.924 | 0.981 |

| **Stroke Volume** | | | | | | | | | | |
| --- | --- | --- | --- | --- | --- | --- | --- | --- | --- | --- |
| Mean ± SD [ml] | 89.5±18.1 | | 87.8±18.5 | 90.2±17.9 | 86.9±17.0 | 84.7±15.0 | 87.4±15.8 | 88.7±15.7 | 88.4±16.3 | 92.4±18.6 |
| Median [ml] | 84.4 | | 81.8 | 84.8 | 83.3 | 83.8 | 84.5 | 83.7 | 85.2 | 92.3 |
| p-value | |  | 0.006 | 0.228 | 0.001 | <0.001 | 0.079 | 0.701 | 0.748 | 0.126 |

| **End-diastolic volume** | | | | | | | | | | |
| --- | --- | --- | --- | --- | --- | --- | --- | --- | --- | --- |
| Mean ± SD [ml] | 127.2±32.1 | | 125.2±32.5 | 127.6±31.8 | 125.5±31.2 | 125.2±30.8 | 125.6±31.1 | 126.6±31.4 | 130.7±33.6 | 134.9±35.2 |
| Median [ml] | 122.1 | | 124.7 | 124.1 | 120.9 | 121.0 | 125.0 | 126.2 | 135.8 | 141.5 |
| p-value | |  | 0.007 | 0.746 | 0.002 | 0.002 | 0.203 | 0.773 | 0.653 | 0.147 |

| **End-systolic volume** | | | | | | | | | | | | | | | | | | |  |  |
| --- | --- | --- | --- | --- | --- | --- | --- | --- | --- | --- | --- | --- | --- | --- | --- | --- | --- | --- | --- | --- |
| Mean ± SD [ml] | | 37.7±21.5 | 37.6±21.5 | | | 37.4±21.4 | | 38.7±21.5 | | 40.1±21.4 | | 38.1±21.9 | | 37.8±21.5 | | 42.3±22.7 | | 42.5±24.0 | | |
| Median [ml] | | 25.9 | 26.1 | | | 25.9 | | 26.4 | | 33.8 | | 26.8 | | 26.7 | | 36.7 | | 35.3 | | |
| p-value |  | | | 0.455 | 0.464 | | 0.002 | | 0.001 | | 0.133 | | 0.683 | | 0.078 | | 1.000 | | |  |

| **Left ventricular outflow tract area** | | | | | | | | | | |
| --- | --- | --- | --- | --- | --- | --- | --- | --- | --- | --- |
| Mean ± SD [ml] | 458±84 | | 458±81 | 458±85 | 452±82 | 448±81 | 457±82 | 459±81 | 467±79 |  |
| Median [ml] | 415 | | 415 | 413 | 412 | 403 | 418 | 420 | 425 |  |
| p-value | |  | 0.920 | 0.863 | <0.001 | <0.001 | 0.709 | 0.777 | 0.064 |  |

| **AVA** | | | | | | | | | | | | | | | | | | |  |  |
| --- | --- | --- | --- | --- | --- | --- | --- | --- | --- | --- | --- | --- | --- | --- | --- | --- | --- | --- | --- | --- |
| Mean ± SD [cm²] | | 0.94±0.23 | 0.92±0.21 | | | 0.94±0.23 | | 0.90±0.23 | | 0.84±0.22 | | 0.91±0.22 | | 0.92±0.22 | |  | |  | | |
| Median [cm²] | | 0.95 | 0.92 | | | 0.97 | | 0.91 | | 0.84 | | 0.94 | | 0.96 | |  | |  | | |
| p-value |  | | | 0.112 | 0.648 | | 0.003 | | <0.001 | | 0.015 | | 0.162 | |  | |  | | |  |

**Patients in atrial fibrillation (n=18)**

| Standard reconstruction | | | Modified reconstructions | | | | | | | |
| --- | --- | --- | --- | --- | --- | --- | --- | --- | --- | --- |
|  | 0.6mm  5%  512x512  FBP | | 0.6 mm  5%  512x512  IR2 | 0.6 mm  5%  256x256  FBP | 0.6 mm  10%  512x512  FBP | 0.6 mm  20%  512x512  FBP | 1 mm  5%  512x512  FBP | 2 mm  5%  512x512  FBP | 5 mm  5%  512x512  FBP | 8 mm  5%  512x512  FBP |
| **Ejection fraction** | | | | | | | | | | |
| Mean ± SD [%] | 64±13 | | 64±13 | 64±13 | 63±13 | 60±12 | 63±13 | 63±13 | 65±11 | 68±9 |
| Median [%] | 65 | | 65 | 65 | 65 | 58 | 64 | 63 | 65 | 68 |
| p-value | |  | 0.248 | 0.317 | 0.003 | 0.002 | 0.081 | 0.227 | 0.439 | 0.306 |

| **Stroke Volume** | | | | | | | | | | |
| --- | --- | --- | --- | --- | --- | --- | --- | --- | --- | --- |
| Mean ± SD [ml] | 84.7±27.0 | | 84.2±27.1 | 85.0±28.0 | 82.1±25.7 | 78.6±27.2 | 82.3±25.8 | 81.8±25.9 | 88.5±26.4 | 97.2±28.9 |
| Median [%] | 76.1 | | 75.2 | 76.0 | 75.0 | 68.2 | 73.0 | 70.7 | 81.5 | 98.3 |
| p-value | |  | 0.182 | 0.723 | <0.001 | 0.002 | 0.133 | 0.286 | 0.733 | 0.061 |

| **End-diastolic volume** | | | | | | | | | | |
| --- | --- | --- | --- | --- | --- | --- | --- | --- | --- | --- |
| Mean ± SD [ml] | 132.9±30.2 | | 132.7±30.4 | 133.7±30.5 | 131.5±29.7 | 130.5±30.3 | 130.7±27.1 | 129.8±27.0 | 135.8±29.2 | 142.4±36.2 |
| Median [ml] | 125.5 | | 127.5 | 126.8 | 122.8 | 120.1 | 127.5 | 123.6 | 121.9 | 138.9 |
| p-value | |  | 0.695 | 0.055 | 0.013 | 0.006 | 0.557 | 0.528 | 0.394 | 0.088 |

| **End-systolic volume** | | | | | | | | | | | | | | | | | | |  |  |
| --- | --- | --- | --- | --- | --- | --- | --- | --- | --- | --- | --- | --- | --- | --- | --- | --- | --- | --- | --- | --- |
| Mean ± SD [ml] | | 48.2±21.4 | 48.5±21.4 | | | 48.7±21.5 | | 49.4±20.9 | | 52.0±19.3 | | 48.4±20.4 | | 48.0±19.5 | | 47.3±18.7 | | 45.2±17.0 | | |
| Median [ml] | | 41.4 | 42.0 | | | 41.7 | | 42.5 | | 49.2 | | 41.3 | | 42.2 | | 40.6 | | 40.3 | | |
| p-value |  | | | 0.136 | 0.072 | | 0.017 | | 0.012 | | 0.500 | | 0.586 | | 0.955 | | 0.460 | | |  |

| **Left ventricular outflow tract area** | | | | | | | | | | |
| --- | --- | --- | --- | --- | --- | --- | --- | --- | --- | --- |
| Mean ± SD [ml] | 517±90 | | 519±88 | 516±89 | 513±88 | 508±88 | 517±89 | 518±92 | 514±97 |  |
| Median [ml] | 515 | | 523 | 511 | 527 | 519 | 527 | 531 | 520 |  |
| p-value | |  | 0.336 | 0.406 | 0.110 | 0.011 | 0.812 | 0.895 | 0.597 |  |

| **AVA** | | | | | | | | | | | | | | | | | | |  |  |
| --- | --- | --- | --- | --- | --- | --- | --- | --- | --- | --- | --- | --- | --- | --- | --- | --- | --- | --- | --- | --- |
| Mean ± SD [cm²] | | 0.88±0.19 | 0.88±0.18 | | | 0.87±0.18 | | 0.84±0.19 | | 0.80±0.17 | | 0.89±0.20 | | 0.88±0.18 | |  | |  | | |
| Median [cm²] | | 0.93 | 0.96 | | | 0.93 | | 0.89 | | 0.85 | | 0.95 | | 0.930 | |  | |  | | |
| p-value |  | | | 0.686 | 0.744 | | 0.008 | | 0.001 | | 0.710 | | 0.794 | |  | |  | | |  |

IR, iterative reconstruction; FBP, filtered back projection; AVA, aortic valve area.
